# Giant Gallstone With Gallbladder Perforation and Hepatic Abscess in an Asian Patient

**DOI:** 10.7759/cureus.39894

**Published:** 2023-06-02

**Authors:** Saroj K Yadav, Mohim Thakur, Ajay Dhiman, Ajeet Kumar, Gopal Sharma

**Affiliations:** 1 Department of General Surgery, All India Institute of Medical Sciences, Bilaspur, Bilaspur, IND; 2 Department of Radiology, All India Institute of Medical Sciences, Bilaspur, Bilaspur, IND

**Keywords:** cholecystectomy, intestinal obstruction, hepatic abscess, gall bladder, giant gallstones

## Abstract

Giant gallstone with a secondary hepatic abscess is a very rare phenomenon. We recently treated a patient with a giant gallbladder (GB) stone of size 11.5 cm with a hepatic abscess who presented with features of an acute abdomen. This was subsequently managed with an open subtotal cholecystectomy and concomitant hepatic abscess drainage. To the best of our knowledge and after a thorough literature search, this is one of the largest reported gall bladder (GB) stones with wall perforation and hepatic abscess in the Asian subcontinent.

## Introduction

Gallstones are very common in southeast Asia, including the Gangetic part of India [1]. Gallstones larger than 5 cm are very rare [2] and are associated with a higher risk of complications like fistulation and hepatic abscess formation [3]. One of the largest gallstones reported in the Asian subcontinent is a 16.8 cm complicating hepatic abscess [4]. Laparoscopic cholecystectomy is the gold-standard surgical approach to treating gallstones in the current era. However, cases of complicated giant gallstones, if approached by a minimally invasive approach, are associated with technical difficulties during the procedure due to dense adhesions and the large size of the stone [5,6]. Herein, we report a case of a complicated giant gallstone managed by open subtotal cholecystectomy and postoperative endoscopic retrograde cholangio-pancreatogram (ERCP) with common bile duct (CBD) stenting. 

## Case presentation

A 57-year-old female with a history of hypertension presented with complaints of fever. The fever persisted over 15 days, was of sudden onset, associated with chills and rigor, and had a maximum recorded temperature of 103°F, with no significant diurnal variation. She reported pain in the upper abdomen for one day, which was sudden in onset, localized to the right upper quadrant, radiating to the right scapular angle, dull aching, moderate to severe in intensity, aggravated by food intake, and relieved by intravenous (IV) analgesics. There were no other bladder or bowel disturbances, loss of weight, or loss of appetite. The patient reported a history of yellowish discoloration of her eyes six months prior, which was not associated with itching or clay-colored stool, which was resolved with medical remedies.

On examination, the patient was hypotensive with tachycardia (104/min), temperature 102°F, and SpO_2_ 98% on room air. She weighed 74 kg with a height of 164 cm and body mass index (BMI) of 27.5. On abdominal examination, there was tenderness and guarding over the upper abdomen. No organomegaly or free fluid was noted.

**Figure 1:**
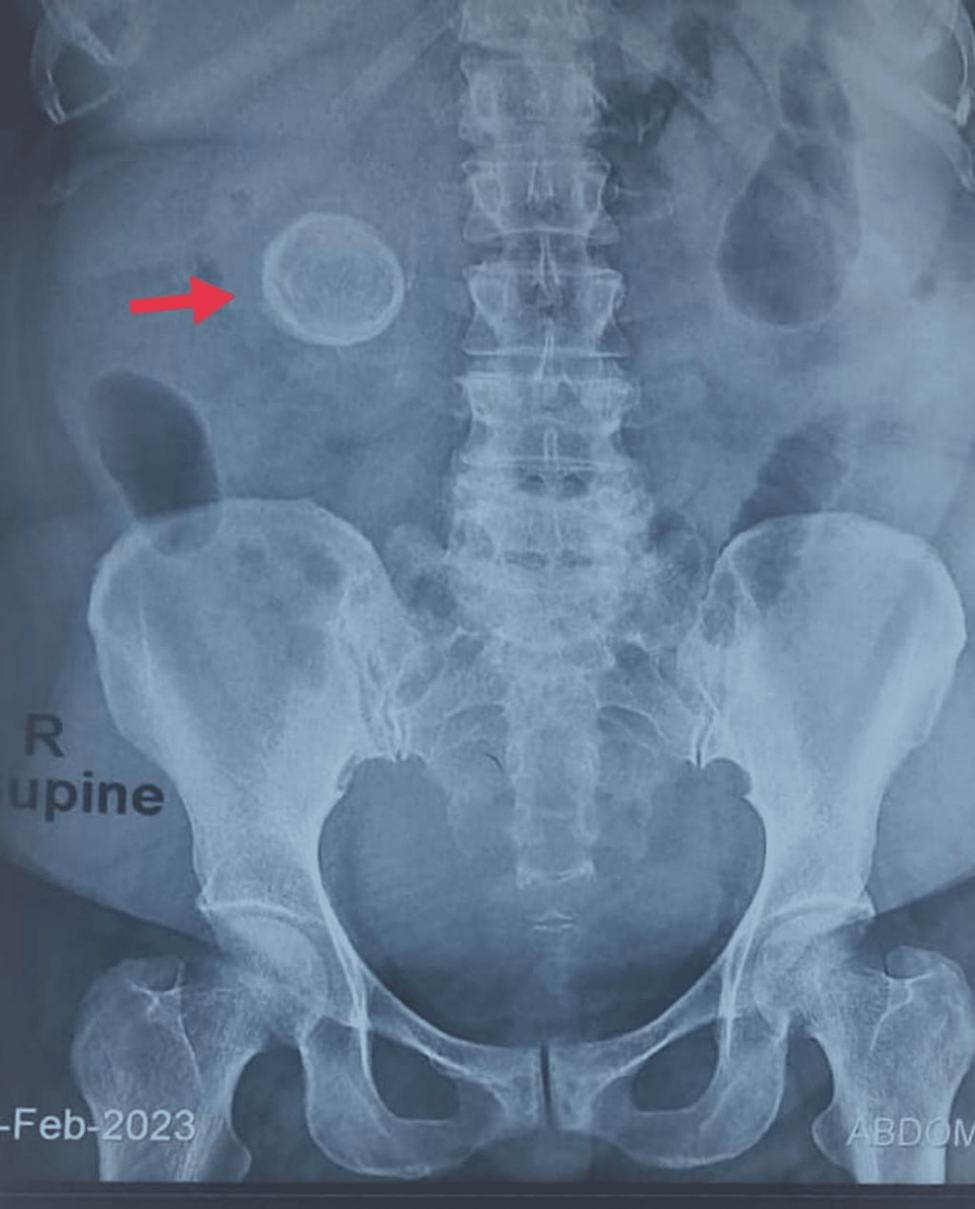
Plain x-ray of the abdomen depicting an oval radio-opaque shadow in the subhepatic region.

After starting IV analgesics and antibiotics, she was evaluated with an ultrasound abdomen that showed a gallstone of size 11.5 cm, impacted in the gallbladder (GB) neck, with discontinuation of the lateral gallbladder (GB) wall facing the liver along with an adjacent collection of 7.6 × 7 cm in the GB fossa. A plain abdominal x-ray showed an oval radiopaque shadow of size 5 × 5 cm in the subhepatic region without any evidence of intestinal obstruction (Figure 1). The supporting laboratory panel depicted increased alkaline phosphatase (ALP) (123 U/L) with normal renal and liver function parameters, normal coagulation, and normal hematological profiles. Per the locoregional protocol for fever evaluation, malaria and dengue serology were found to be negative.

**Figure 2:**
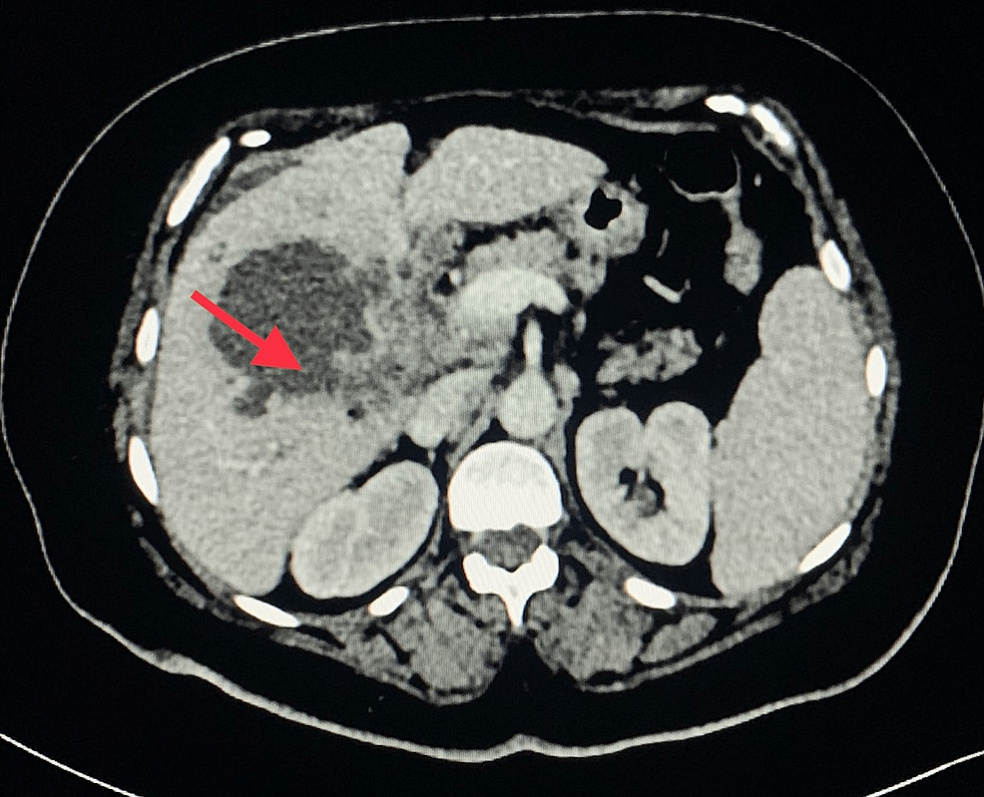
CT axial view showing the rent in the GB wall with a 6 × 7 cm hepatic abscess in liver segment IV. CT: computerized tomography, GB: gallbladder.

**Figure 3:**
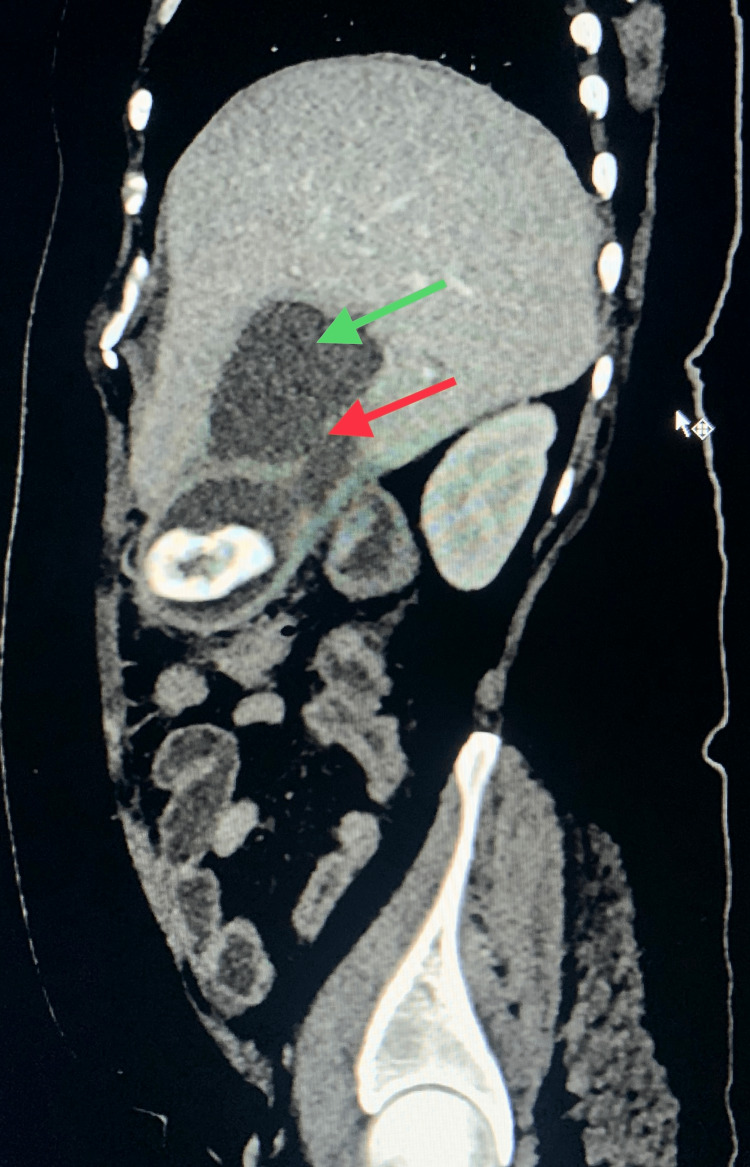
CT sagittal view showing a large gallstone with a defect in the GB wall (red arrow) and an adjacent hepatic segment IV abscess (green arrow) CT: computerized tomography, GB: gallbladder.

**Figure 4:**
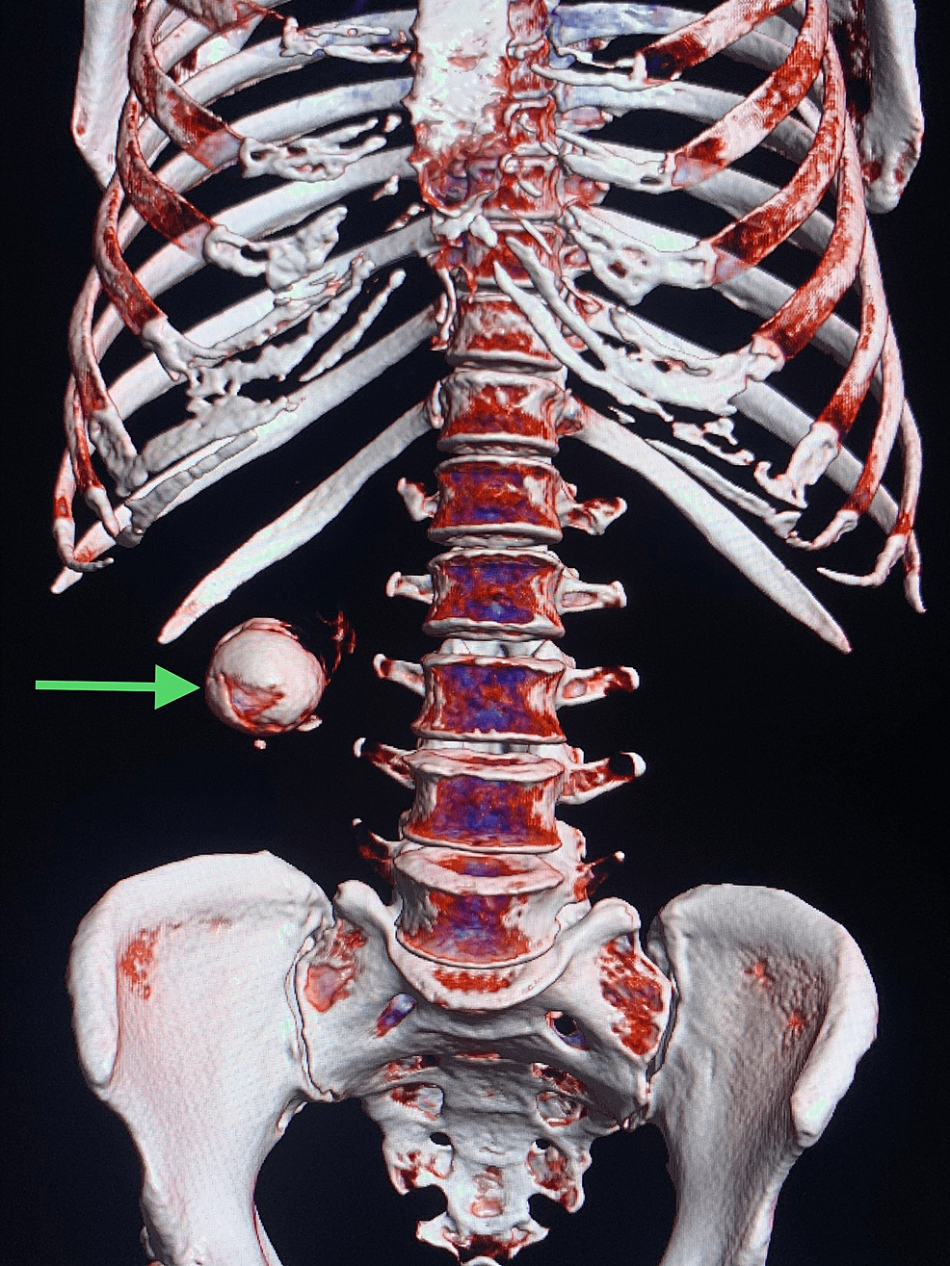
Reconstructed three-dimensional CT image showing a giant gallstone. CT: computerized tomography.

The patient was further evaluated with cross-sectional contrast-enhanced computerized tomography (CT) imaging on the same day that showed the GB as being over-distended and thick-walled (5.3 mm), with large radiopaque calculus (11.5 × 6 cm), with a breach in the lateral wall of the GB towards the surface of the liver (Figure 2), and with a loculated collection of 6 × 7 × 6.1 cm in the GB fossa region (Figure 3). The reconstructed CT image shows the GB stone (Figure 4). The common bile duct (CBD) was prominent with a 7 mm caliber.

**Figure 5:**
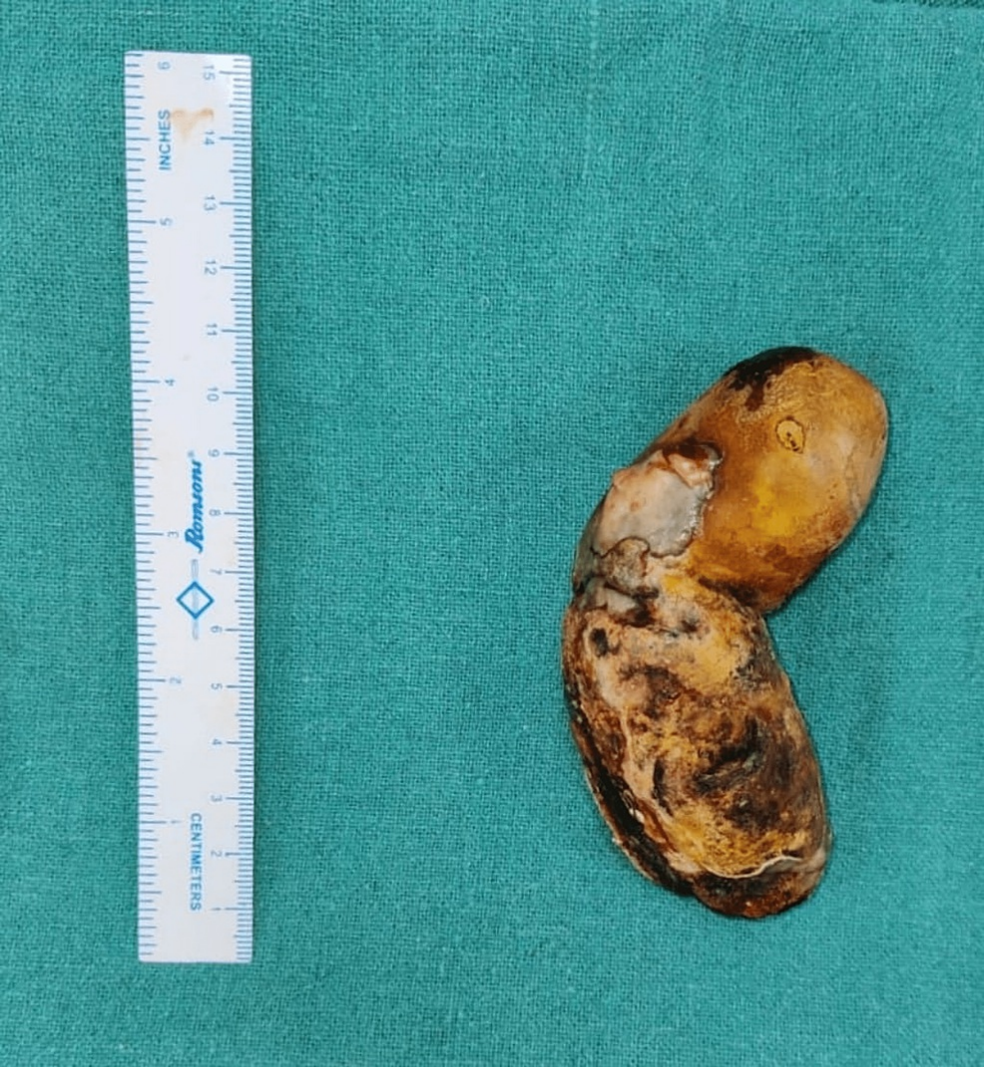
Giant gallstone of size 11.5 × 5.5 cm.

**Figure 6:**
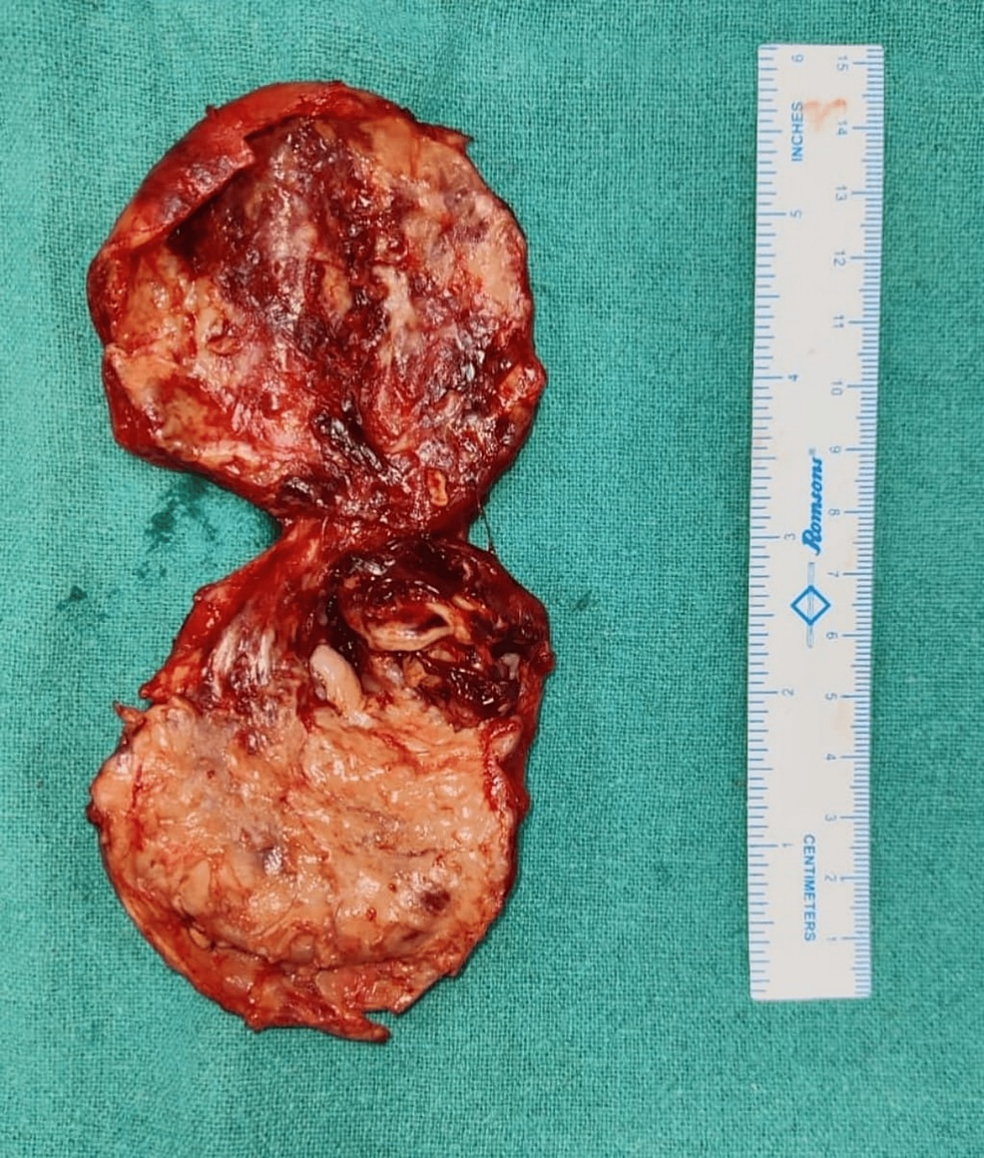
Resected gall bladder part laid open.

With the working diagnosis of GB perforation, the patient was initially managed conservatively, and subsequently, surgical intervention was planned because of worsening abdominal symptoms and signs. Given the complicated nature of the giant gallstone, an open approach was considered for the patient's safety and the predicted difficulty of laparoscopy. After a generous Kocher’s incision, intraoperatively 100 ml of ascitic fluid was drained, and the note was made of omental clumping near the subhepatic region. A bilio-purulent collection of approximately 200 ml was drained from the liver near the GB fossa. The GB was inflamed, thick, and large, approximately 15 cm × 12cm, with a frozen Calot's triangle. No significant enlarged lymph nodes were noted around the GB, CBD, or portal vein. The GB fundus was incised transversely, and a stone of size 11.5 cm was removed with difficulty (Figure 5). Because of the frozen Calot's triangle, its dissection was avoided, and only the part of the GB body with the fundus was resected (Figure 6). The mucosa of the GB remnant was burned with electrocautery, and a reconstituting subtotal cholecystectomy was performed. A subhepatic 32 Fr drain (La-Med Healthcare Pvt Ltd., Faridabad, India) was placed upon completion of the procedure.

Postoperatively, the patient was hemodynamically stable and afebrile. Abdominal signs settled over the next postoperative day, with minimal serous drain output. The patient developed a low-output biliary fistula postoperatively, which was successfully managed with ERCP CBD stenting using a 10 Fr biliary pigtail stent (Biorad Medisys Pvt Ltd., Pune, India). Drain output became nil a day after ERCP stenting. The subhepatic drain was removed subsequently, and the patient was discharged with advice for stent removal after six weeks.

The histopathological examination of the resected GB part depicted features suggestive of xanthogranulomatous cholecystitis (XGC). The patient was advised for regular follow-up with interval imaging. 

## Discussion

Gallstones disease (GSD) is more common in females, especially during their fertile periods. This is possibly due to increased estrogen turnover, with secondary effects on cholesterol efflux into the bile and GB motility [7]. In India and South Asia, the ratio of males: females having GSD is 1:2.5 as per various studies [1]. Age-wise, the frequency of GSD increases as age advances [5,7,8]. The associated comorbidities affecting the occurrence of GSD are dyslipidemia, diabetes mellitus, non-alcoholic liver steatosis, and obesity [8].

The etiopathogenesis of gallstones results from an irregularity in the typical relationship among the major constituents of bile, including cholesterol, phospholipids, and bile acids [5,7,8]. There are three main steps in gallstone formation: bile saturation, crystallization, and enucleation [7]. Once the cholesterol level increases, saturated vesicles are formed that initiate the nucleation of cholesterol monohydrate crystals, which form the core of a cholesterol stone [7]. Chronic cholecystitis aids in lithogenesis via excessive mucin secretion and rapid cholesterol crystal formation [7,9]. The contents of mixed and pigmented stones are cholesterol with bile pigment or mainly bile pigment, respectively. This basic mechanism of stone formation remains the same for all sizes of stone. Predisposing factors for the formation of giant gallstones have not been elucidated in the literature.

Clinically, GSD can have a broad spectrum of presentations, ranging from asymptomatic cases to biliary colic, acute and chronic cholecystitis, GB perforation, choledocholithiasis, and acute pancreatitis [7,8]. Approximately, 60-80 % of GSD cases are asymptomatic, whereas 20% of patients will eventually develop gallstone-related complications at an incidence of 1-4% annually [5,7,8]. Approximately 10-15% of patients usually present with acute calculus cholecystitis as the first clinical entity [2,7]. Another very rare complication of GSD is gallstone ileus, which may occur in less than 0.5% of patients. The diagnosis is often delayed because of the non-specific presentation clinically and radiologically [10].

GB perforation might be documented in 2-11% of patients with acute calculus cholecystitis, resulting from ischemia and necrosis [9,11]. The GB fundus remains the most common site to be affected because of its vulnerable blood supply [3,11]. However, in this index case, the perforation was at the right lateral wall of the GB, where sealing with the omentum and bowel is easy, leading to pericholecystic fluid accumulation.

Laparoscopic cholecystectomy has been the standard of care for GSD, which can be achieved in almost 96% of cases [2,5,7]. There is debate about whether to consider giant gallstones for the laparoscopic approach or the classical open approach. In this instance, the open subtotal cholecystectomy was performed because of the large size of the gallstone, GB perforation/hepatic abscess, frozen Calots, and the hemodynamic status of the patient.

## Conclusions

A giant gallstone presenting with gall bladder perforation and a hepatic abscess is a rare entity that needs early diagnosis and immediate surgical intervention. Although a gallbladder perforation is usually managed with a conservative approach comprising a tube cholecystostomy or percutaneous drainage followed by interval laparoscopic/open cholecystectomy, an immediate surgical intervention either laparoscopic or open, would be more promising in hemodynamically unstable patients. Although laparoscopy is the standard of care for gallbladder stones, an open approach with total or subtotal cholecystectomy would be more appropriate and safer for complicated giant gallstones presenting with a frozen Calot’s triangle and compromised hemodynamic status. Early surgical drainage and cholecystectomy are the cornerstones of management for such complicated case. This index case is the largest reported complicated gallstone in the Asian subcontinent and was managed by open subtotal cholecystectomy.
